# P02.49. Dynamical system approach for CAM research

**DOI:** 10.1186/1472-6882-12-S1-P105

**Published:** 2012-06-12

**Authors:** A Ahn, P Wayne, G Yeh, Y Liu, C Peng

**Affiliations:** 1 Massachusetts General Hospital Martinos Center for Biomedical Imaging, Charlestown, USA; 2 Brigham Women's Hospital, Boston, USA; 3 Beth Israel Deaconess Medical Center, Boston, USA; 4 DynaDx Corporation, Mountain View, USA

## Purpose

Complementary and alternative medicine (CAM) therapies often approach health and disease from a holistic viewpoint that is congruent with the contemporary systems biology framework. Conventional biomedical markers and outcomes typically focus on individual components of whole human systems, while ignoring important nonlinear interactions among different components of the system. New analysis tools that are specifically designed to help understand the impact of CAM interventions from a systems biology framework are needed.

## Methods

With support from an NCCAM SBIR grant, we enhanced the nonlinear dynamics software package called DataDemon (DynaDx Inc.) by modifying the user interface and creating new modules to increase accessibility and applicability to CAM researchers. Based on a dynamical system approach, DataDemon allows the study of physiologic fluctuations with measures that reflect emergent properties of integrative systems. Modifications in Phase I included Hilbert-Huang transform, multiscale entropy analysis (a measure of system complexity), enhanced wavelet analyses, among others. New software was shared with a diverse sample of CAM researchers to evaluate usability, clarity, and application to a variety of physiological data.

## Results

By applying the modified software to existing CAM research data, we successfully obtained non-linear measures of heart rate dynamics and center of pressure control following tai-chi mind-body interventions, performed wavelet analyses of arterial pulse waves following deep breathing, and determined complexity of electrical potential measurements in acupuncture meridians. Overall, preliminary feedback suggested good functioning of the software with a clear and friendly user interface. Suggestions for improvement included generalized dynamical coupling analysis for multi-systems interactions.

## Conclusion

DataDemon is a promising software package that uses a new dynamical system approach to provide quantitative measures for CAM research and interventions. Further development will focus on broader sampling of CAM users, new interfaces (e.g. functional magnetic resonance imaging, ultrasound), systems coupling analyses, and richer tutorial programs to make the software more widely accessible.

